# Determinant of Dynamics and Interfacial Forces in Ultraprecision Machining of Optical Freeform Surface through Simulation-Based Analysis

**DOI:** 10.3390/mi14122228

**Published:** 2023-12-12

**Authors:** Ali Khaghani, Atanas Ivanov, Kai Cheng

**Affiliations:** Department of Mechanical and Aerospace Engineering (MAE), Brunel University London, London UB8 3PH, UK; atanas.ivanov@brunel.ac.uk (A.I.); kai.cheng@brunel.ac.uk (K.C.)

**Keywords:** micromachining, ultraprecision machining, single point diamond turning machining, interfacial forces, multi-body dynamics, optical freeform surfaces, slow tool servo

## Abstract

This study delves into the intricacies of ultraprecision machining, particularly in the context of machining optical freeform surfaces using Diamond Turning Machines (DTMs). It underscores the dynamic relationship between toolpath generation, hydrostatic bearing in DTMs, and the machining process. Central to this research is the innovative introduction of Metal Matrix Composites (MMCs) to replace the traditional materials used in designing linear bearings. This strategic substitution aims to dynamically enhance both the accuracy and the quality of the machined optical freeform surfaces. The study employs simulation-based analysis using ADAMS to investigate the interfacial cutting forces at the tooltip and workpiece surface and their impacts on the machining process. Through simulations of STS mode ultraprecision machining, the interfacial cutting forces and their relationship with changes in surface curvatures are examined. The results demonstrate that the use of MMC material leads to a significant reduction in toolpath pressure, highlighting the potential benefits of employing lightweight materials in improving the dynamic performance of the system. Additionally, the analysis of slideway joints reveals the direct influence of interfacial cutting forces on the linear slideways, emphasising the importance of understanding and controlling these forces for achieving higher-precision positioning and motion control. The comparative analysis between steel and MMC materials provides valuable insights into the effects of material properties on the system’s dynamic performance. These findings contribute to the existing body of knowledge and suggest a potential shift towards more advanced precision forms, possibly extending to pico-engineering in future systems. Ultimately, this research establishes a new standard in the field, emphasising the importance of system dynamics and interfacial forces in the evolution of precision manufacturing technologies.

## 1. Introduction

Diamond turning, a precision-engineered subset of ultraprecision machining, is increasingly recognized for its efficacy in producing optical freeform surfaces. The novel ultraprecision toolpath planning method for slow tool servo diamond turning optimises freeform optical surface machining by considering surface curvature and differential geometry for enhanced surface quality and uniformity [1]. This technique stands out for its integration of fast tool servo (FTS) and slow tool servo (STS) methodologies, which are key to achieving high accuracy in complex surface geometries. Such surfaces find extensive applications in a variety of industries including optics, automotive, electronics, aerospace, and biomedical engineering, reflecting their growing importance in modern precision engineering [2,3,4]. The process begins with a CAD/CAM system generating a toolpath trajectory, a critical step where the trajectory may either precisely mimic the freeform surface geometry or incorporate calculated adjustments to correct any deviations in the surface form [5,6]. A novel stochastic toolpath strategy significantly improves surface quality in freeform component machining, while concurrent research on hydrostatic bearing sliders reveals critical assembly error tolerances, vital for large-scale structural applications [7,8]. This initial phase sets the foundation for the precision and quality of the final machined product.

In ultraprecision machining, particularly in the context of diamond turning, the differentiation between FTS and STS modes presents distinct challenges and opportunities. Each mode employs unique strategies for material removal, impacting the achievable surface finish. Despite their advantages, obtaining the optimal surface finish is a persistent challenge, raising questions about the limits of current methodologies [8,9,10]. The sector’s evolution is driven by escalating demands for precision and productivity, especially in the machining of freeform surfaces. Addressing these demands calls for a nuanced understanding of a range of factors: the dynamics of the machining process, the properties and behaviour of the materials involved, the mechanical stiffness and friction characteristics of the system, the precision of tooling, and the accuracy of servo systems. Nevertheless, an advanced tracking error prediction model for ultraprecision machines addresses the critical impact of cutting force disturbances on servo control systems and the resulting profile accuracy of machined parts [11]. These elements do not operate in isolation but interact in complex ways, influencing the performance and capabilities of ultraprecision machining systems. Furthermore, hydrostatic bearings, employing incompressible fluids under high pressure, present multiple benefits such as enhanced damping, substantial static stiffness, effective pressure distribution despite surface flaws in bearings, and superior load capacity [12]. Even with imperfect surfaces, these bearings maintain error motion ratios under 100 nm [13]. Nonetheless, at higher velocities, their performance is constrained by increased dynamic friction. In ultraprecision machining (UPM) spindles, particularly at high speeds, managing power loss due to this friction is crucial. STS systems frequently use hydrostatic bearings, leveraging their considerable stiffness and damping for load bearing and achieving satisfactory linear accuracy during machining’s backward and forward motions [14].

This study builds upon the findings of earlier research [15,16,17], extending the exploration into the realm of multi-body dynamics in ultraprecision machining, with a focus on optical freeform surfaces. Previous studies have significantly advanced the field by developing new methodologies for effective toolpath generation for freeform surfaces and enhancing the general dynamic effects in an ultraprecision machining system. Notably, these advancements include the introduction of Metal Matrix Composite (MMC) materials as a replacement for linear bearings, a change that has shown promise in improving machining dynamics. However, while these studies have laid a solid foundation, they have not fully explored the specific dynamic effects of these advancements on optical freeform surfaces. In particular, the impact of MMC material replacement on the toolpath dynamics and the resultant quality of the machined optical freeform surfaces has not been thoroughly investigated. This research aims to fill this gap by meticulously examining how the integration of MMC materials influences the toolpath, interfacial forces, and overall surface quality in the ultraprecision machining of optical freeform surfaces. Our study extends beyond the existing methodologies, offering new insights into the optimisation of ultraprecision machining processes for the production of high-quality optical components.

MMCs are hypothesized to significantly influence the dynamics of the machining process, particularly in how the cutting tool interacts with the workpiece. This research investigates the role of interfacial cutting dynamics, a critical factor that has received limited attention in previous studies. By examining the interaction between the tool and workpiece through an interconnected multi-body elements, the study aims to unlock new levels of precision in machining. The incorporation of MMC, noted for its superior damping characteristics and higher resonant frequencies, is expected to mitigate adverse interfacial forces that can degrade surface quality. This approach is complemented by the use of slow tool servo technology which enables precise control over tool movements, essential for achieving the refined surface finishes required in high-precision optical applications. The overarching goal of this research is to advance the field of precision engineering, specifically targeting the achievement of pico-precision levels in the fabrication of optical freeform surfaces. The insights gained are anticipated to guide the development of next-generation ultraprecision machining systems, setting new benchmarks for precision in the production of high-quality optical components.

## 2. Dynamic Effects in Ultraprecision Machining with Slow Tool Servo Mode

The STS methodology facilitates a synchronised interplay between slide and spindle movements in diamond turning machines, thereby enabling the generation of freeform surfaces without the necessity for supplementary tool axes [18,19]. Nonetheless, there exists a pressing requirement to enhance the dynamics governing these motions. Furthermore, there is a compelling need for a scientific elucidation of the interactions at the interface between the cutting tool tip and the workpiece surface. Such advancements are integral for elevating the precision and productivity of ultraprecision machining systems. Operating in STS mode on a diamond turning machine necessitates the presence of frictionless axes, pinpoint control mechanisms, and due consideration of various factors. These include encoder resolution, thermal stability, trajectory formulation, data acquisition, and overall system rigidity. Within the STS operational framework, a meticulous analysis of the positioning loop and associated dynamics proves to be of paramount importance. This is largely attributable to the direct influence exerted by the topological characteristics of the freeform surface on both tool velocity and acceleration. Achieving a high bandwidth for the positioning loop, coupled with a nuanced understanding of the interrelationships among surface geometry, actuation mechanisms, and control algorithms, is indispensable for optimising system dynamics [16].

Traditional approaches to ultraprecision toolpaths are often inadequate at capitalising on the system dynamics that are modulated by the freeform surface in question. A nuanced comprehension of the interfacial dynamics between the tool tip and the workpiece surface, as well as the dynamics inherent within the machining loop, is indispensable for the evolution of ultraprecision machining systems that are both more precise and more efficient. In summary, the process of generating tool paths for freeform surface machining involves designing the tool path based on surface specifications, selecting appropriate machining parameters, considering dynamic cutting forces, conducting tool interference analysis, analysing tool axis motion, developing numerical models for surface generation, and performing tool compensation analysis. Following these steps ensures high precision and productivity in freeform surface machining.

While existing methods for toolpath generation can produce cutting paths for freeform surfaces in ultraprecision machining, they often overlook the dynamic and kinematic effects impacting both the tool and surface characteristics. To attain greater precision and efficiency, further research is needed to explore the inherent relationship between these dynamic influences and the resultant surface finish. Tool selection in STS machining is pivotal, particularly in relation to the surface’s curvature and sagittal dimensions. The tool nose radius should match the surface’s maximum and minimum curvature to avoid interference. Tool included and front clearance angles must be smaller than the surface’s maximum curvature angle. Dynamic and kinematic effects of the tool on surface geometry should be considered, especially for surfaces with larger sagittal curvatures.

## 3. Advancing Precision and Associated Implementations

The current passive positioning control techniques in ultraprecision machining fall short when striving for elevated levels of precision accuracy. To overcome these limitations and attain higher resolution such as pico-precision, real-time oversight and regulation of both tool and workpiece positions are imperative. Considering the multi-body dynamics, the three-axis ultraprecision machining procedure can be conceptualised as a rigid sliding wedge, bolstered by a preloaded spring and propelled by magnetic forces [17].

**Figure 1:**
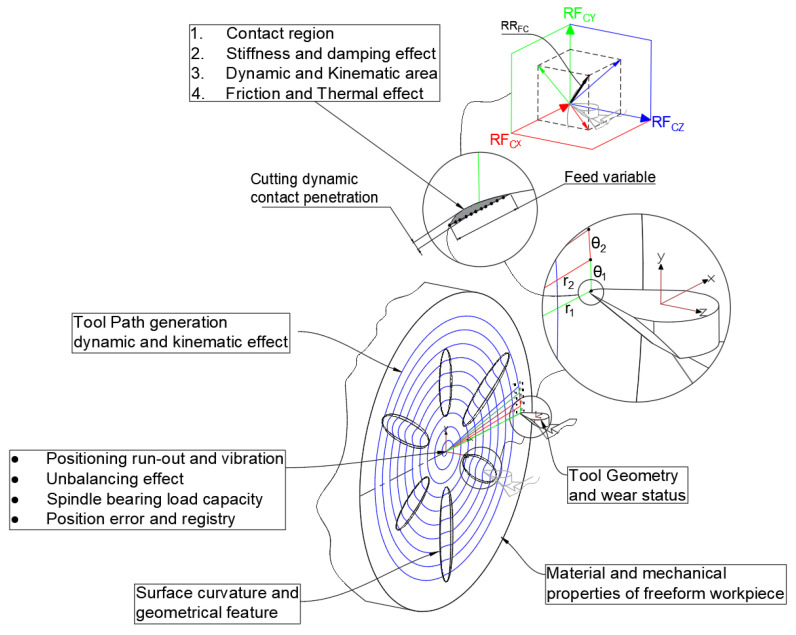
Dynamic effect factors in positioning controls [16,17].

According to [17], the interfacial force $F_{C}y$, $F_{C}x$ and $F_{C}z$ exhibits varied reactions at different points on the freeform surface due to its curvatures. The nonlinear behaviour of these forces at the centre of slide mass emphasises the mutual influence between cutting forces and slide performance. It is important to measure forces at each toolpath point to enhance control system capability, enabling a shift from semi-closed loop to fully closed loop. Figure 1 emphasises the dynamic effects and factors that need to be considered in the monitoring and control of tool and workpiece positioning.

Notwithstanding the progress achieved in open-loop and closed-loop control systems within the realm of ultraprecision machining, as highlighted in preceding research, the deployment of MMC in such systems is yet to be thoroughly examined. This is particularly pertinent for the machining of optical freeform surfaces which are characterised by their diverse and complex curvatures. Although modern ultraprecision machining systems are outfitted with high-resolution encoders capable of refined kinematic positioning and advanced closed-loop control taking into account the dynamic interactions at the interface between the tool tip and workpiece, the unique dynamic effects induced by the application of MMC to surfaces with variable curvatures remain underexplored. The present study is committed to addressing this gap by assessing the impact of MMC integration on the dynamics of closed-loop control systems, with a specific focus on the intricate topographies associated with optical freeform surfaces. A comprehensive understanding of these dynamics is essential to drive the development of ultraprecision machining systems forward, ensuring that they can satisfy the exacting precision requirements of the production of next-generation optical components.

## 4. Dynamics Simulation Using ADAMS

To address the research gap in the literature regarding STS technology in ultraprecision machining, two simulation models were created using ADAMS software. As is evident, machining freeform surfaces is influenced by the workpiece’s surface topology and geometrical curvature. Therefore, to study the dynamics effects with a high level of accuracy, an ultraprecision machining component is required to conduct the simulation. For this particular purpose, an existing industrial-level diamond turning machine was modelled in CAD software Solidworks 2019 and imported into ADAMS version 2022.2 for dynamic and kinematic analysis in this study.

**Figure 2:**
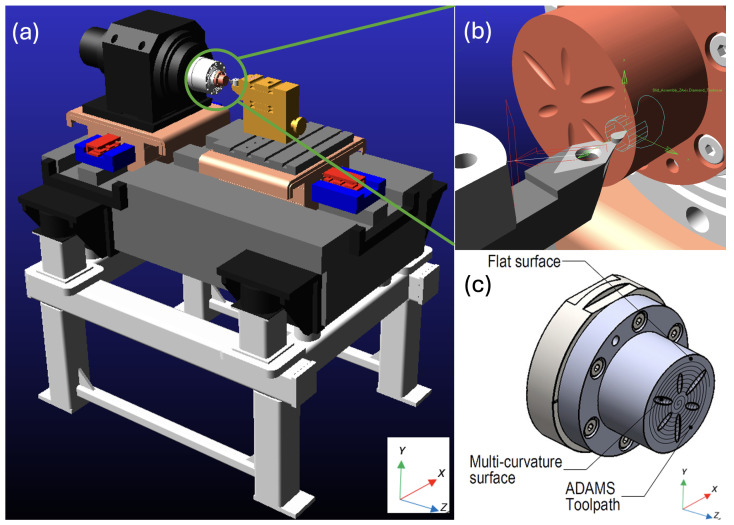
ADAMS model for hydrostatic bearing slideway analysis: (**a**) imported CAD model of UPM (Nanotech 250UPL); (**b**) multi-curvature workpiece; (**c**) workpiece with toolpath generated by ADAMS.

The first model utilised a standard prismatic linear connection joint for the slideways in both the X and Z axes with alloy steel material, while the second model used a similar joint constraint for the slides with MMC material to maintain linear motion for hydrostatic bearing between the base and carriage planar surfaces. Figure 2 illustrates the ADAMS model and the multi-curvature workpiece designed for this study.

### 4.1. Numerical Analysis and FEM Result Discussion

To initiate the research, 3D CAD models were imported into ADAMS, and a toolpath generation (TPG) process was implemented using the multi-body dynamic methodology that recently developed [16]. Table 1 provides the ADAMS solver parameter data for TPG and the numerical setup used in the study.

**Figure 3:**
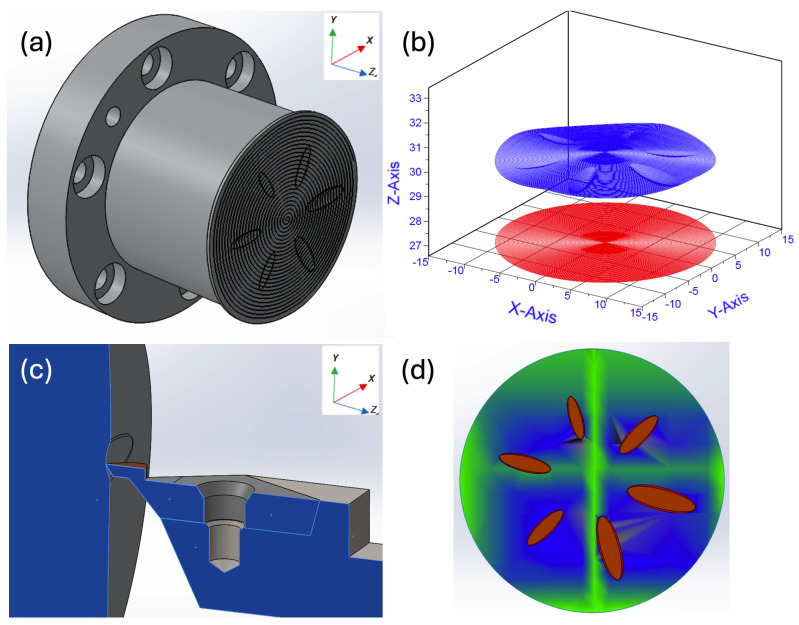
Freeform surface with large curvature: (**a**) freeform workpiece; (**b**) toolpath generated by ADAMS/solver; (**c**) freeform workpiece with grooves; (**d**) surface curvature analysis.

Figure 3a illustrates the 3D toolpath generated by ADAM and Figure 3b displays the final 2D and 3D toolpath curve generated in a vector type of x, y, and z directions in the Cartesian plane. Red colour illustrates the 2D projected toolpath and blue represents the 3D Toolpath. A different approach was adopted to investigate the dynamic impact on a freeform surface with more significant sag. Figure 3c shows a freeform surface with several grooves with a higher curvature value. The maximum curvature value in the grooves was obtained from the curvature analysis, as depicted in Figure 3d. After running the simulation, a precise toolpath was generated based on the tool and surface geometry, as illustrated in Figure 3b. The multi-body dynamic method enables the calculation of dynamical effects such as contact force, velocity, acceleration, moment force, and displacements within the toolpath generation.

### 4.2. Toolpath and Interfacial Dynamic Force Effects

To validate the impact of the interfacial force on the toolpath, two models with different material densities, alloy steel and MMC, were analysed. The assumption was that changing the mass would result in a significant dynamic response in the system and the reaction forces in Cartesian coordination at the tooltip would increase the impact pressure in the toolpath. The preliminary analysis confirms this hypothesis. The material properties for MMC (Al2024) with an average particle size of 3 μm, as assigned to the z-axis, are provided according to [15,20].

**Figure 4:**
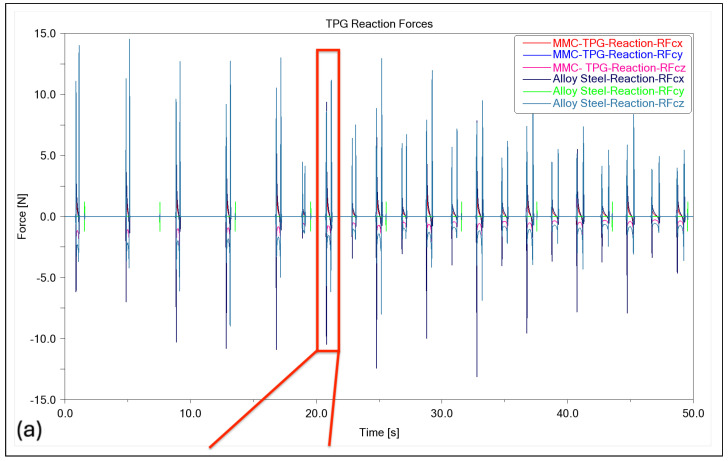

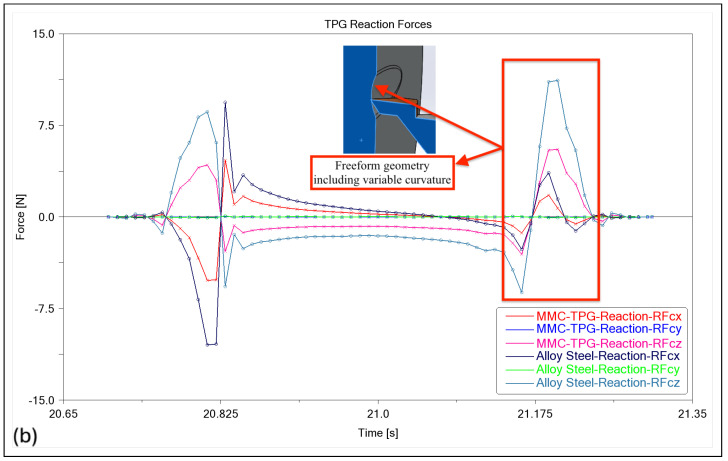
Interfacial forces in freeform toolpath joints: (**a**) full step time; (**b**) partial step time.

The graph presented in Figure 4a,b demonstrates the impact of surface curvature on the reaction force at the tooltip, which is connected to the toolpath. Observing only the specific time range highlighted in red frame box in Figure 4b, which represent the partial toolpath through this specific groove in a freeform surface, a peak analysis for max positive and negative force can initially assist in understanding the effects of dynamics forces through the material removal process. In the provided analysis, the interfacial force data for two materials, alloy steel and MMC, across three different directions, are denoted as x, y, and z. The dataset contained distinct time-series data for each material–direction combination, resulting in six unique force curves. The primary focus was on identifying the peak forces, both the maximum positive and the maximum negative values, for each curve. The results revealed that for alloy steel, the peak forces in the x-direction reached a maximum of 3.5521 N and a minimum of −2.5264 N, in the y-direction a maximum of 0.0696 N and a minimum of −0.1203 N, and in the z-direction a maximum of 11.2769 N and a minimum of −6.1355 N. Similarly, for MMC, the x-direction showed a maximum force of 1.8425 N and a minimum of −1.1967 N, the y-direction a maximum of 0.0591 N and a minimum of −0.0890 N, and the z-direction a maximum of 5.5783 N and a minimum of −3.0329 N. These peaks are indicative of the most significant positive and negative forces each material experiences in each direction. The analysis reveals that the reaction forces in the x and z directions of the slides (RFcx and RFcz), as shown in Figure 1, possess higher magnitudes. Furthermore, the findings indicate that utilising MMC material with reduced mass leads to a decrease in pressure at the cutting toolpath, resulting in reduced reaction forces at the tooltip during the cutting process. This reduction in reaction forces can contribute to the enhanced dynamic performance of the system in a more robust and feasible manner.

### 4.3. Interfacial Force Comparison Analysis

**Figure 5:**
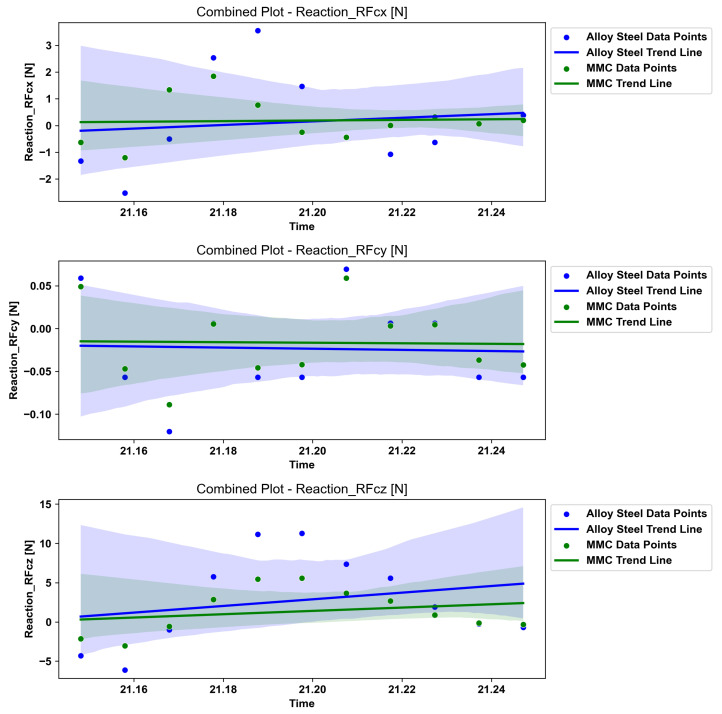
Interfacial Force Comparison, MMC vs. Alloy Steel.

The analysis of the force data, particularly through scatter plots, provided compelling evidence of the reduction in forces when using MMC material compared to alloy steel. This was quantitatively evident across the X, Y, and Z dimensions, but most notably in the RFcy dimension, where the method’s sensitivity to small interfacial forces was clearly demonstrated. Figure 5 illustrates the result for further qualitative analysis using the scatter trend statistics method to understand the impact of employing different material on the toolpath. The findings of this analysis demonstrate three major criteria as follows:

- Force Reduction in MMC:
  Across all dimensions, MMC consistently exhibited lower peaks in force magnitude compared to alloy steel. This was quantitatively apparent from the scatter of data points, where MMC’s force values were often lower than those for alloy steel at similar time points. This trend suggests that MMC, due to its material properties, is more effective in reducing the magnitude of forces experienced during the simulated conditions.
- RFcy Dimension—Small Interfacial Forces:
  The analysis in the RFcy dimension was particularly revealing. Here, the scatter plot showed that MMC not only experienced lower force magnitudes compared to alloy steel but also captured very small interfacial forces. The data points for MMC in this dimension were tightly clustered, indicating less variability and a remarkable ability to respond to very small force changes. This capability to detect and respond to extremely small interfacial forces in the RFcy dimension underscores the potential of this method for future applications requiring pico-precision. It highlights the sensitivity and precision inherent in the measurement technique, making it suitable for advanced applications where detecting subtle force changes is crucial.
- Implications for Future Precision Applications:
  The observed reduction in forces with MMC and the method’s ability to capture very small interfacial forces, especially in the RFcy dimension, hold significant implications for future precision engineering applications. The results suggest that MMC could be a preferred material choice in scenarios where minimizing force impact is critical. Additionally, the method’s sensitivity in detecting small interfacial forces indicates its potential utility in developing and testing materials and systems designed for ultraprecision machining technology.
  Nevertheless the quantitative analysis not only demonstrated a clear reduction in forces with the use of MMC but also showcased the method’s capability of capturing very small interfacial forces, particularly in the RFcy dimension. These findings are pivotal in positioning MMC as a material conducive to reduced force environments and affirming the method’s suitability for pico-precision applications in future technological advancements.

**Figure 6:**
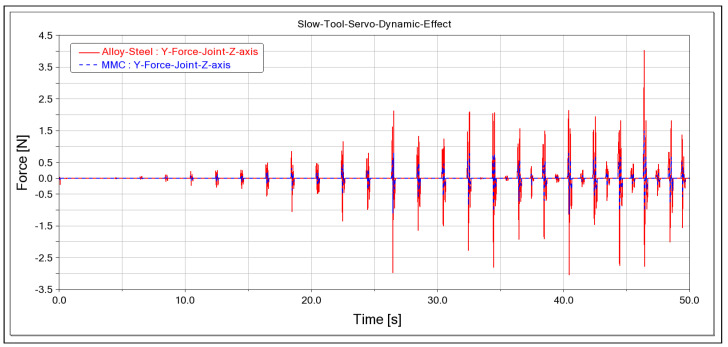
Dynamic force comparison in both MMC and alloy steel, prismatic joint y-direction reaction force.

This study also analysed the dynamic forces of the system, including the contact force at the tool nose, the force at the prismatic joint, and the toolpath mate reaction forces. The dynamic impact of the system was compared between MMC and alloy steel materials. The graph presented in Figure 6 shows the dynamic forces calculated in the y-direction of the prismatic joint, according to [17], for both MMC and alloy steel materials. The y-direction in the z-axis is the vector where the cutting force is applied, making it crucial to analyse the dynamic impact in this direction. The result indicates that the peak value calculated for MMC is lower than that of alloy steel in the prismatic joint of the z-axis slideway. This suggests that the mass has a direct effect on the performance of the system’s motions.

**Figure 7:**
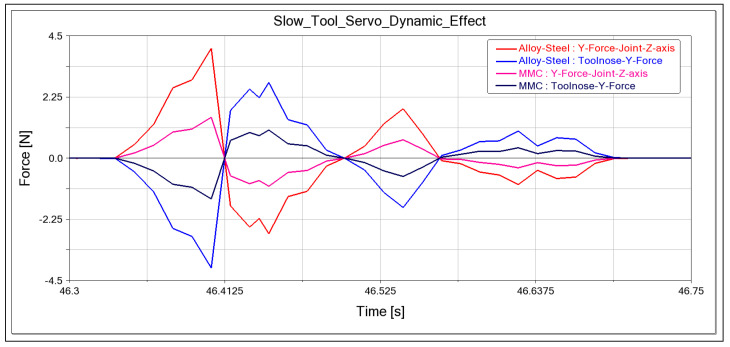
Calculated dynamic force in both MMC and alloy steel, tool nose y-direction reaction force vs. prismatic joint y-direction reaction force.

Moreover, this study investigates the influence of forces exerted on the tool tip on the linear slideways, an effect that is anticipated in ultraprecision machining. The simulation models a continuous sliding motion, characterised by a dynamic static friction coefficient, which was set at 0.03 for the hydrostatic bearings. It is noted that the tool nose is subject to varying forces in the y-direction, arising from contact forces and friction. Figure 7 indicates that the force vector at the tool nose is in the opposite direction to the reactive force at the joint in the z-axis, suggesting a negative value for the force reaction in the y-direction, a finding further supported by Figure 4b.

A significant observation from the study is that the reaction force values for MMC are lower than those for alloy steel, underscoring the material’s potential impact on machining dynamics. The directionality of forces at the tool nose, opposing the reaction forces at the prismatic joint, highlights the crucial role of dynamic interfacial forces in affecting the accuracy of toolpath joints, which is essential for the precise creation of freeform optical surfaces. In practical applications, hydrostatic bearing constraints are typically configured as planar joints, with fixed planar surfaces on the base body parallel to the movable surfaces on the carriage. The study suggests that further investigation is necessary to examine the influence of toolpath pressure and the corresponding reactions within the system, as well as to assess the dynamic effects that may necessitate the use of a planar joint over a prismatic joint. This points to the potential need for experimental validation to confirm the simulation findings.

## 5. Conclusions

This study reveals that the proposed method of generating toolpaths for ultraprecision machining of freeform surfaces using multi-body dynamics analysis has significant implications for achieving higher precision in the machining system. By considering the interfacial cutting forces at the tooltip and workpiece surface, the method provides valuable insights into the dynamic behaviour of the machining process. The simulation-based approach using ADAMS allows for a thorough investigation of the interfacial cutting forces and their impacts on the machining system. This provides a deeper understanding of the complex interactions between the tool and workpiece, contributing to the advancement of ultraprecision machining techniques. Furthermore, the comparative analysis conducted on two different materials, steel and MMC, examined the effects of material properties on the dynamic performance of the slideways. The reduction in toolpath pressure observed when using MMC material highlights the potential benefits of employing lightweight materials to enhance the overall system performance.

The analysis of the scatter plots examining the forces experienced by alloy steel and MMC across the RFcx, RFcy, and RFcz dimensions revealed significant insights:

- The plots highlighted a notable reduction in force magnitudes when using MMC compared to alloy steel, demonstrating MMC’s superior performance in minimizing force impact under simulated conditions.
- In the RFcy dimension, the analysis was particularly revealing, showcasing the method’s capability to detect very small interfacial forces, thereby underscoring its potential for applications requiring pico-precision.
- The data points did not exhibit a clear linear trend across any of the dimensions, indicating the presence of fluctuating force patterns and the absence of a consistent directional trend in the forces experienced over time.
- This variability in the forces, coupled with the reduced force impact observed with MMC, highlights the material’s suitability for high-precision applications where controlling and minimizing force magnitudes are crucial.

In conclusion, this study introduces an innovative way to enhance the dynamics of ultraprecision machining using a novel method of toolpath generation, highlighting the importance of considering interfacial cutting forces and material properties. The simulation-based analysis provides valuable insights into the dynamics and performance of the machining system, paving the way for advancements in precision manufacturing.

## Figures and Tables

**Table 1:** Numerical motion study and analysis data [16].

**Analysis Data**	**Value**	**Units**
Rotary motor speed	30	RPM
Feed rate (x-axis)	0.01	mm/s
Initial force	1	N
Dynamic friction	0.25	-
Static friction	0.3	-
Elastic impact stiffness	100,000	N/mm
Max Damping	50	N/(mm/s)
Penetration	0.0	mm
**Integrator**	**Value**	**Units**
Frames per second	360	-
3D Contact Resolution	High	-
Accuracy	0.0000001	-
Integrator Type	WSTIFF	-
Max Iteration	25	-
Initial Integrator Step Size	0.00001	-
Min Integrator Step Size	0.000001	-
Max Integrator Step Size	0.01	-
Jacobian Re-evaluation	Every Iteration	-

## Data Availability

The data in this study is unavailable due to data privacy and restrictions.
